# Clustering Users Based on Hearing Aid Use: An Exploratory Analysis of Real-World Data

**DOI:** 10.3389/fdgth.2021.725130

**Published:** 2021-09-03

**Authors:** Alessandro Pasta, Tiberiu-Ioan Szatmari, Jeppe Høy Christensen, Kasper Juul Jensen, Niels Henrik Pontoppidan, Kang Sun, Jakob Eg Larsen

**Affiliations:** ^1^Department of Applied Mathematics and Computer Science, Technical University of Denmark, Kongens Lyngby, Denmark; ^2^Demant A/S, Smørum, Denmark; ^3^Eriksholm Research Centre, Oticon A/S, Snekkersten, Denmark

**Keywords:** data logging, user clustering, ensemble classification, hearing aid use amount, hearing aid use patterns, hearing aids, personalization

## Abstract

While the assessment of hearing aid use has traditionally relied on subjective self-reported measures, smartphone-connected hearing aids enable objective data logging from a large number of users. Objective data logging allows to overcome the inaccuracy of self-reported measures. Moreover, data logging enables assessing hearing aid use with a greater temporal resolution and longitudinally, making it possible to investigate hourly patterns of use and to account for the day-to-day variability. This study aims to explore patterns of hearing aid use throughout the day and assess whether clusters of users with similar use patterns can be identified. We did so by analyzing objective hearing aid use data logged from 15,905 real-world users over a 4-month period. Firstly, we investigated the daily amount of hearing aid use and its within-user and between-user variability. We found that users, on average, used the hearing aids for 10.01 h/day, exhibiting a substantial between-user (SD = 2.76 h) and within-user (SD = 3.88 h) variability. Secondly, we examined hearing aid use hourly patterns by clustering 453,612 logged days into typical days of hearing aid use. We identified three typical days of hearing aid use: full day (44% of days), afternoon (27%), and sporadic evening (26%) day of hearing aid use. Thirdly, we explored the usage patterns of the hearing aid users by clustering the users based on the proportion of time spent in each of the typical days of hearing aid use. We found three distinct user groups, each characterized by a predominant (i.e., experienced ~60% of the time) typical day of hearing aid use. Notably, the largest user group (49%) of users predominantly had full days of hearing aid use. Finally, we validated the user clustering by training a supervised classification ensemble to predict the cluster to which each user belonged. The high accuracy achieved by the supervised classifier ensemble (~86%) indicated valid user clustering and showed that such a classifier can be successfully used to group new hearing aid users in the future. This study provides a deeper insight into the adoption of hearing care treatments and paves the way for more personalized solutions.

## Introduction

It is estimated that, globally, 430 million people have disabling hearing loss, i.e., a hearing loss greater than 35 decibels (dB) in the better hearing ear (1). By 2050 over 700 million people are expected to have disabling hearing loss (1). Untreated hearing loss has repercussions at an individual level. It is associated with poorer cognitive and psychological status, resulting in increased risk of depression, dementia, falls, and quality of life (2–4). Hearing loss negatively impacts education, employment, and household income (1, 5). Additionally, untreated hearing loss has a negative impact on society and the economy. Older adults with untreated hearing loss experience higher health care costs and utilization patterns compared with adults without hearing loss (4). The World Health Organization (1) estimates that untreated hearing loss poses an annual global cost of US$ 980 billion, including health sector costs, costs of educational support, loss of productivity, and societal costs.

The adoption of hearing aids (HAs) has been shown to have a positive impact on the quality of life of users (6, 7) and to mitigate the effect on their household income (5). The success of HA provision as a treatment for hearing loss depends on the fact that the patient is provided with a favorable change in their condition, but also on the patient compliance with the intervention program (8). Perez and Edmonds (8) conducted a systematic review to identify and evaluate how studies have measured and reported the use of HAs in older adults. A limited number of studies (5 out of 64) were found to assess HA use based on objective measures, such as data logging and battery consumption. Most of the studies assessed HA use through self-reported measures, such as standardized questionnaires, custom questionnaires, interviews, and diaries. However, self-reported measures have been shown to diverge from objective measures, leading to inaccurate and overreported HA use (9–12). In addition to avoiding such recall bias, objective data logging enables measuring HA use with a greater temporal resolution and longitudinally (13). The widespread adoption of smartphones among older adults (14) and the introduction of smartphone-connected hearing aids make it possible to objectively assess the HA use of a larger number of users than ever before (15).

When evaluating HA usage, the amount of HA use time is commonly regarded as an indicator of treatment success (16) and frequently investigated (9, 12, 17–19). Although the amount of HA use time generally correlates with HA satisfaction (20), this metric might not provide a complete picture. Indeed, frequent use does not necessarily equate with benefit (21). A previous study found that some HA users reported low HA use time and high HA satisfaction, while other users reported high HA use time and low HA satisfaction (22). Furthermore, HA use time provides information about how much the HA has been used during the day, but it is not informative of when and how the HA has been used. For instance, two users might exhibit the same amount of use time (e.g., 8 h), but use the HAs at different times of the day (e.g., from 8:00 to 16:00 and from 15:00 to 23:00) or in different ways (e.g., on-off usage or continuous usage). For these reasons, in addition to the amount of HA use time, other patterns of HA use should be analyzed (11). However, methods possessing low temporal resolution (e.g., self-reports or accumulated use time across a day or a week) do not account for the hourly and daily variability in HA use. Smartphone-connected HAs enable continuous data logging, thereby making it possible to assess the hourly HA use and more accurately identify recurrent use patterns.

Additionally, the HA industry is currently predominantly accommodating for the average user (23). However, the amount of HA use varies widely among HA users (9, 19, 24). Similarly, the pattern of HA use has been reported to vary among HA users. Laplante-Lévesque et al. (11) clustered 171 HA owners, showing that 57% had, on average, a continuous HA use during the day, while 43% had an on-off HA use. A qualitative study (16) reported that optimal HA use depends on the individual needs of the HA owners and does not necessarily correspond to wearing the HAs most of the time. Some HA users reported that they do not depend on their HAs and that they experience situations which they can successfully attend without HAs. Therefore, it is of interest to objectively measure and investigate the HA use of a large number of HA owners, in order to identify and quantify different types of users based on their HA use patterns. This potentially enables gaining deeper insight into the adoption of hearing care treatments and paves the way for more personalized solutions (25).

Finally, when comparing users based on their HA use, the average individual use is usually considered. This means that the within-user variability in HA use is often disregarded (11, 19, 24). However, HA users might exhibit different HA use patterns from one day to another and two HA users with the same average use might behave differently. For instance, two users might exhibit the same average amount of use time throughout the logged days (e.g., 8 h), but one might use the HA constantly (e.g., 8 h each day) while the other might exhibit more variation among the days (e.g., alternating days with 2 and 14 h of use). Therefore, when comparing users based on HA use, it is desirable to adopt a metric that goes beyond the average use per user and that considers the within-user variability.

In this study, we analyze the objective HA use data logged from 15,905 real-world users over a 4-month period. Firstly, we investigate the daily amount of HA use and its within-user and between-user variability. Secondly, we examine HA use hourly patterns by clustering the 453,612 logged days to identify typical days of HA use. Thirdly, we explore the usage patterns of the HA users and investigate whether we can cluster the users based on how they used the HAs during the logged days. When performing the user clustering, instead of representing each user by her average HA use pattern, we consider the proportion of time spent in each of the typical days of HA use. Finally, we validate the HA user clustering by training a supervised classifier to predict the cluster to which each user belongs.

## Materials and Methods

### Participants and Apparatus

This study used data from a large-scale internal database, which logs the HA use of HA owners who have signed up for the HearingFitness™ feature (25) *via* the Oticon ON™ smartphone app. The participants were the users of Oticon Opn™ hearing aids who used the HearingFitness™ feature for at least 10 days in the period between June and September 2020.

### Data and Data Pre-processing

When the HAs are connected to the smartphone, the HearingFitness™ feature logs timestamped data about the HA use every 10 min. Based on HA use time (i.e., inferred from time counters embedded in the HAs) and connection status, an estimate of hourly HA use (measured in min/h) is computed. For binaural HA users, if the HA use amount was different between the right and left ear, this study selected the larger value, as done by Laplante-Lévesque et al. (11) and Walker et al. (27). If temporary disconnections occur, replacements for the missing data are injected by analyzing the time counters embedded in the HAs. When the disconnected use is full-time use (e.g., 120 min of use during 2 h of disconnection), the HA use during disconnection is simply assigned to the hours of disconnection. When the disconnected use is on-off use (i.e., not full-time use), the minutes of use are evenly distributed among the hours of disconnection (e.g., 60 min of use during 2 h of disconnection result in 30 min/h use for 2 h). The raw data set comprised 1,160,520 days of HA use from 32,216 users. In order to preserve representative patterns of HA use throughout the day, days with on-off use during temporary disconnections longer than 2 h were removed. Additionally, 12,876 days with more than 60 min/h were removed. This is likely a consequence of use time estimation when disconnections occur. Moreover, since this study focuses on analyzing HA use, only days with at least 60 min of HA use were included. Furthermore, only data related to HA use between 6:00 and 23:59 were included. Finally, to ensure that users' behavior was inferred from a representative sample of days, only users with at least 10 days of HA use were included. The cleaned data set comprised 453,612 days of HA use from 15,905 users (28.5 days per user on average).

### Data Analysis

**Figure 1:**
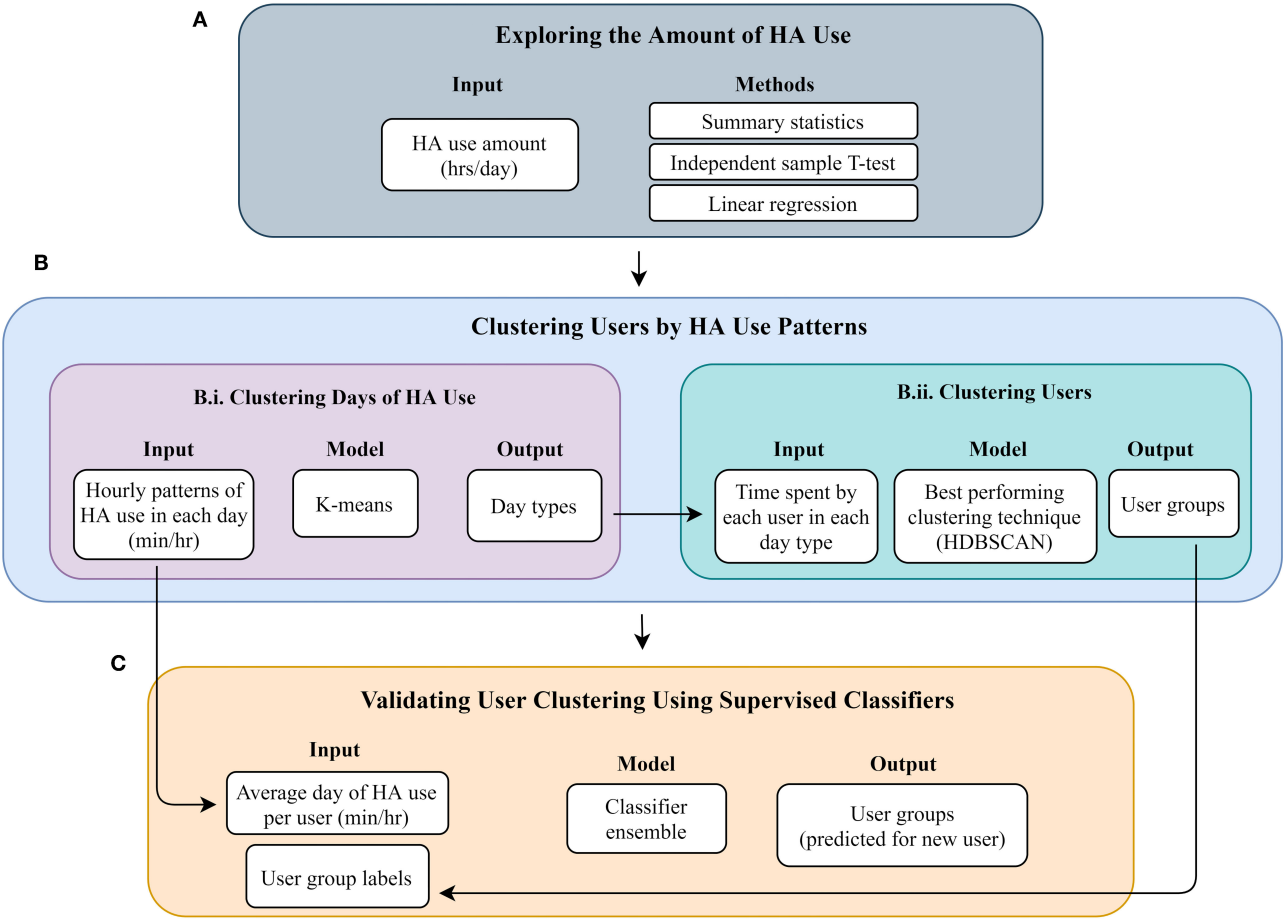
Flow of data analysis. **(A)** Exploring the amount of HA use. **(B)** Clustering users by HA use patterns. **(C)** Validating user clustering using supervised classifiers.

Figure 1 provides an overview of the flow of the data analysis we performed, presenting the main steps undertaken. More details on each step are provided below.

#### Exploring the Amount of Hearing Aid Use

We explored the amount of HA use (measured in hours/day), by computing summary statistics of the 453,612 logged days (mean, SD) and of the amount of HA use for each user (mean, between-user SD, quartiles). Furthermore, we analyzed the within-user daily variability (SD) in HA use amount. Independent sample *t*-tests were performed to compare the within-user SD of medium users (i.e., users with average HA use amount between *Q*_1_ and *Q*_3_) with that of light and heavy users (i.e., users with average HA use amount, respectively, below *Q*_1_ and above *Q*_3_). Cohen's *d* was computed to assess the magnitude of the differences (45). A polynomial linear regression was fitted to model the relationship between average amount of HA use per user (x) and within-user SD (y).

#### Clustering Days of Hearing Aid Use

We examined patterns of HA use by clustering the 453,612 logged days into typical days of HA use. The input data consisted of a 453, 612 × 18 matrix


$$\begin{array}{l} A_{r\times c}=A_{453612\times 18}=\left[\begin{array}{c} a_{11}\cdots a_{1c}\\ \vdots \;\ddots \;\vdots \\ a_{r1}\cdots a_{rc} \end{array}\right]\\ \;=\left(a_{ij}\right)\in \left[0,60\right]\;i=1,\ldots ,r;j=1,\ldots ,c \end{array}$$

where each row *i* represents a day of HA use, each column *j* represents an hour of the day (from 6 to 23) and *a*_*ij*_ is the amount of HA use (from 0 to 60 min) in the day *i* and hour *j*. The *k*-means clustering technique was applied (28), since it is suitable for large data sets. *K*-means aims to partition the observations in *k* clusters by minimizing the within-cluster variance (i.e., square Euclidean distances). The *k*-means++ initialization algorithm (29) was applied, which seeks to spread out the *k* initial clusters to avoid poor approximation. The optimal value of *k* was determined using the elbow method (30), which aims to select a number of clusters so that adding another cluster does not substantially increase the explained variation. The resulting clusters were evaluated by conducting a Silhouette analysis (31), which aims to evaluate the between-clusters dispersion (i.e., separation) and the within-cluster dispersion (i.e., cohesion). A Silhouette Coefficient (ranging from −1 to +1) was calculated for each observation and constitutes a measure of how similar an observation is to its own cluster compared to the next nearest cluster. Furthermore, principal component analysis was performed to visualize the observations in a lower dimensional space. Subsequently, for each cluster, we identified and removed observations that were abnormally distant from the other observations (i.e., below *Q*_1_ − 1.5 · *IQR* and above *Q*_3_ + 1.5 · *IQR*). This was done in order not to include days of HA use that exhibited atypical patterns and were not well-represented by the cluster centroids. The association between the type of day of HA use and the day of the week was tested by performing a χ^2^ test of independence and computing Cramer's V. The clustering and related analyses were performed in Python, using the scikit-learn library (32).

#### Clustering Users

We explored the behavior of HA users by clustering the 15,905 users based on the proportion of time spent in each of the typical days of HA use. The input data consisted of a 15, 905 × *c* matrix


$$\begin{array}{l} B_{r\times c}=B_{15905\times c}=\left[\begin{array}{c} b_{11}\cdots b_{1c}\\ \vdots \;\ddots \;\vdots \\ b_{r1}\cdots b_{rc} \end{array}\right]\\ \;=\left(b_{ij}\right)\in \left[0,1\right]\;i=1,\ldots ,r;j=1,\ldots ,c \end{array}$$

where each row *i* represents a HA user, each column *j* represents one of the *c* typical days of HA use (referring to the clusters found *via* section Clustering Days of Hearing Aid Use) and *b*_*ij*_ is the proportion of days belonging to day type *j* for user *i*. Different clustering techniques were evaluated: *k*-means with *k*-means++ initialization algorithm, Hierarchical Agglomerative Clustering (HAC) with Ward's method, HAC with Pearson correlation and average linkage method, and Hierarchical Density-Based Spatial Clustering (HDBSCAN). HAC (33) initially treats each observation as a cluster and then builds nested clusters by successively merging pairs of the most similar clusters. HDBSCAN (34) groups observations that are in a dense region while marking the observations in sparse regions as noise. It expands on a different density-based technique, DBSCAN (35), by converting it into a hierarchical clustering technique, followed by extracting a flat clustering based on cluster stability. For *k*-means, the optimal value of clusters was determined using the elbow method (30). For HAC, the optimal value of clusters was determined using the dendrogram. HDBSCAN, instead, infers the optimal number of clusters based on the data. For each clustering technique, three internal validation metrics were computed: Silhouette score (31), Caliñski-Harabasz score (36), and Davies-Bouldin score (37). The Caliñski-Harabasz score is defined as a ratio of separation and cohesion. The Davies-Bouldin score measures the average similarity between each cluster and its most similar one, by comparing the distance between clusters with the size of the clusters themselves. Based on the three metrics, the best performing clustering technique was selected. The clustering was performed in Python, using the scikit-learn (32) and hdbscan (38) libraries.

#### Validating User Clustering Using Supervised Classifiers

We validated the HA user clustering by training an ensemble of supervised classifiers to predict the cluster label for individual users based on the average day of HA use for each user. The input data for classification consisted of a 15, 905 × 18 matrix:


$$\begin{array}{l} D_{r\times c}=D_{15905\times 18}=\left[\begin{array}{c} d_{11}\cdots d_{1c}\\ \vdots \;\ddots \;\vdots \\ d_{r1}\cdots d_{rc} \end{array}\right]\\ \;=\left(d_{ij}\right)\in \left[0,60\right]\;i=1,\ldots ,r;j=1,\ldots ,c \end{array}$$

where each row *i* represents the average day of a HA user, each column *j* represents the hour of the day (from 6 to 23) and *d*_*ij*_ is the average amount of HA use (from 0 to 60 min) for user *i* in the hour *j*. This data was further split into separate training and testing data sets with an 80/20 split. To reduce bias (39), three classifiers were chosen from different families: multiclass logistic regression (regression), an XGBoost classifier (decision trees) (40) and a fully connected (FC) neural network classifier (41). The following individual parameters were chosen:

- Multiclass logistic regression: L2 penalty and “newton-cg” solver.
- XGBoost: estimators = 100, max depth = 5, gamma = 0, alpha = 0.1.
- FC neural network: four-layer network (128-64-32-4), ReLU activation, cross-entropy loss, Adam optimizer; trained for 25 epochs.

In order to reduce bias (39), a classification ensemble was defined, which assigns each user to a group by majority voting between the three classifiers. In cases where no majority could be defined, the group was decided by the best performing individual classifier. Two metrics were used to gauge each model's performance: accuracy, and Area Under the Receiver Operating Characteristic (ROC-AUC). Accuracy is obtained by calculating the ratio of correct test predictions to the total amount of samples in the testing set. ROC-AUC helps visualize the relationship between sensitivity (i.e., True Positive Rate) and specificity (i.e., False Positive Rate) for a binary classification problem. The ROC-AUC value ranges from 0 to 1 and represents the ability of a classifier to distinguish between classes at various thresholds. If the current classification task operates with more than two classes (i.e., multiclass classification), the individual classes are first binarized. The score of the individual classes is calculated, then a micro-average is computed by aggregating the contributions of all classes to compute the average metric. Finally, a macro-average is calculated by computing the metric independently for each class and then calculating the average. The training and evaluation of the supervised classification ensemble was performed in Python, using scikit-learn (32), XGBoost (40), PyTorch (42), Yellowbrick (43), and scikit-plot libraries (44).

## Results

### Exploring the Amount of Hearing Aid Use

**Figure 2:**
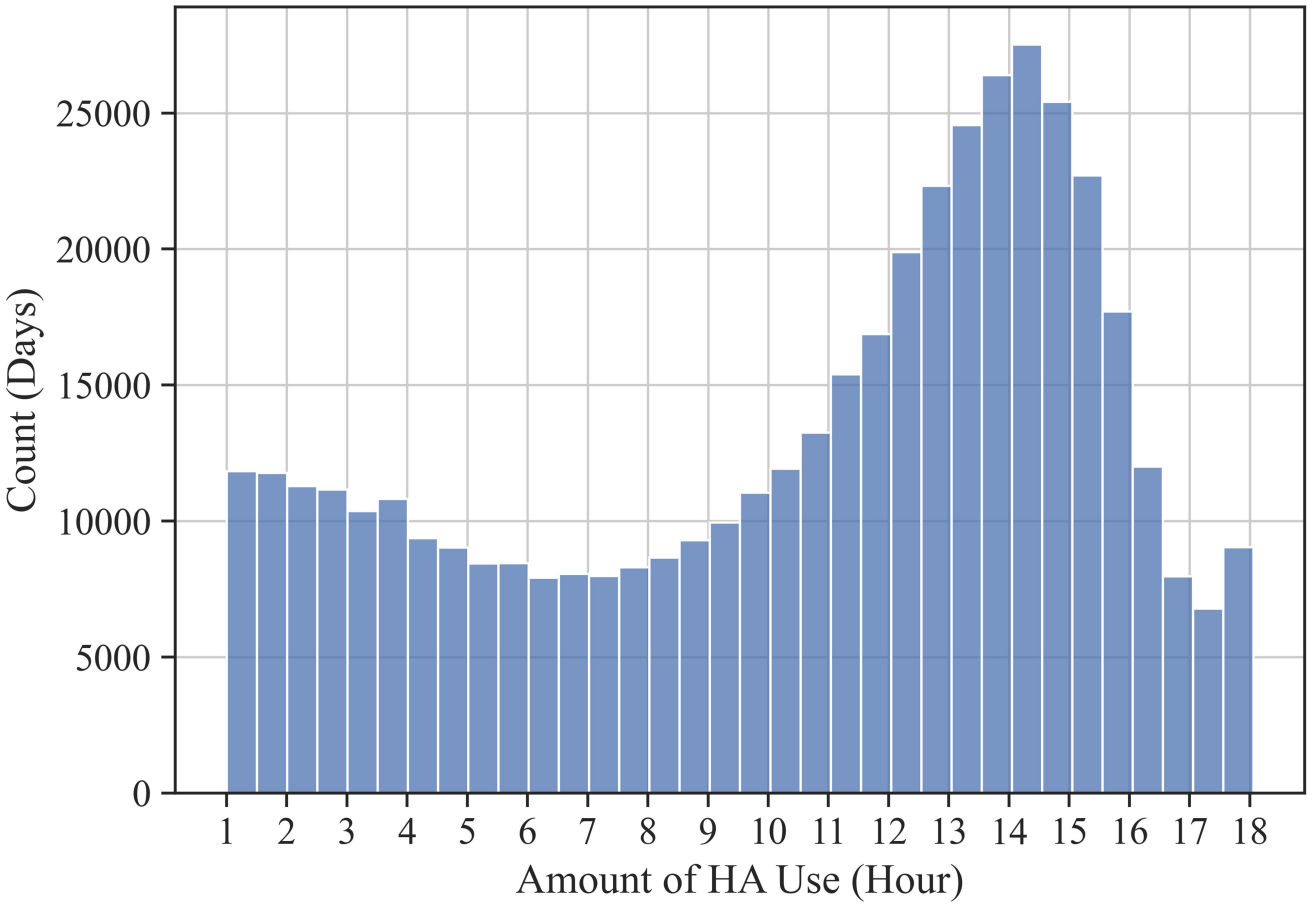
Count of logged days by the amount of HA use time. Due to the data cleaning criteria (i.e., only days with at least 1 h of HA use were included; only data related to HA use in the 18 h between 6:00 and 23:59 were included), the amount of HA use (x-axis) ranges from 1 to 18 h.

The clean data set comprised 453,612 days of HA use from 15,905 users. The amount of HA use, defined as hours of HA use per day, was assessed to describe usage. Figure 2 shows the frequency distribution of HA use amount during the pooled logged days. The data represents the HA use in days of connected use. On average, a day of HA use amounted to 10.55 h. However, the days were not normally distributed around the mean. The amount of HA use widely varied throughout the logged days (SD = 4.71 h), with a mode around 14 h of use and a smaller peak around 1 h of use.

**Figure 3:**
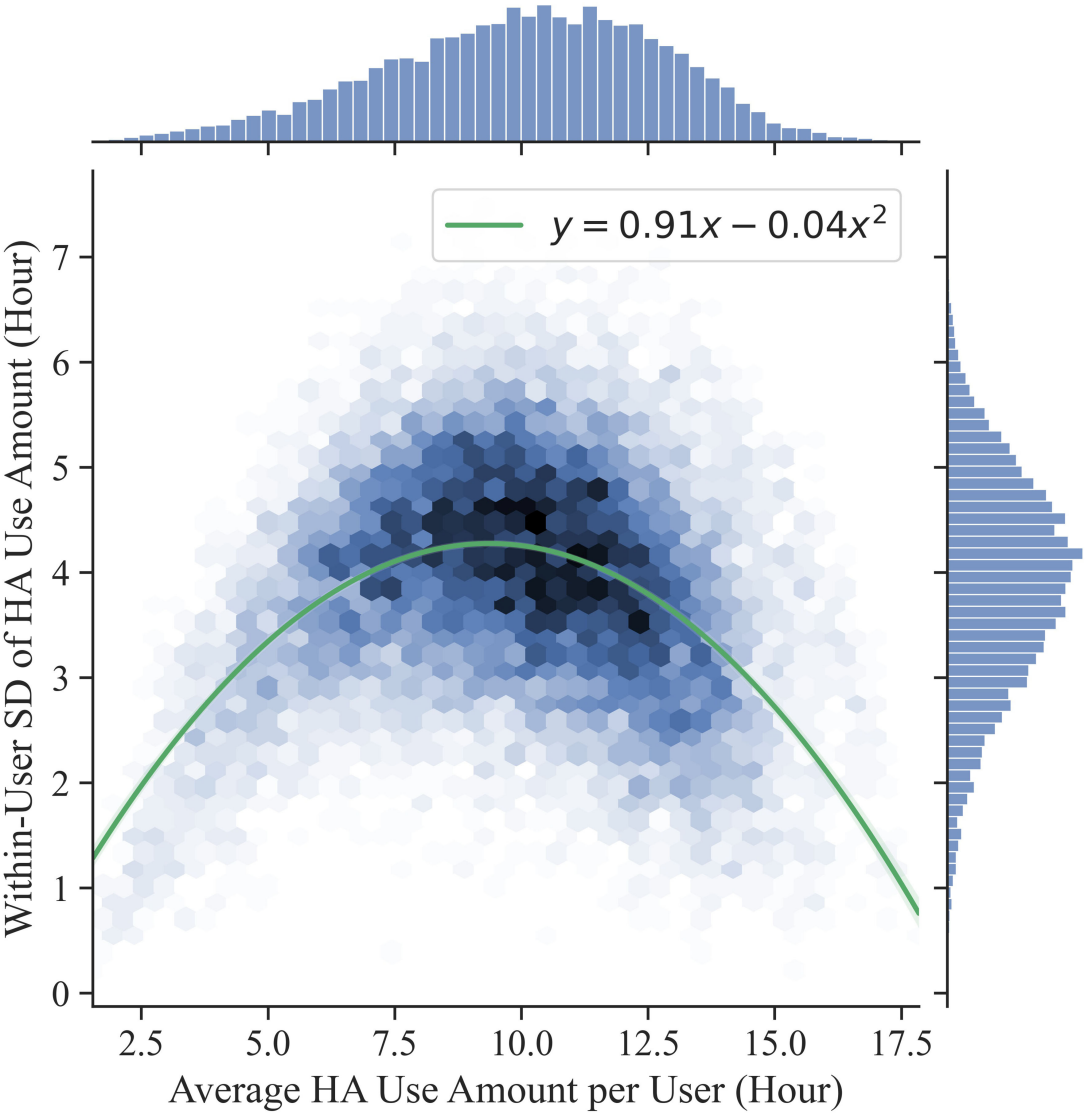
Distribution of users by their average amount of HA use and their variability among the logged days (i.e., within-user SD). A second order linear regression model (line ± 99% confidence interval) was fitted to the data to model the relationship between average HA use (x) and within-user SD (y).

On average, 28 days (SD = 18 days) were logged for each user. We investigated the extent to which users used the HAs differently among each other (i.e., between-user variability) and the extent to which the same user used the HAs consistently throughout the logged days (i.e., within-user variability). For each user, the average amount of HA use and the within-user standard deviation (SD) among the days of HA use were computed. Figure 3 shows the distribution of the 15,905 HA users. We firstly investigated the between-user variability in the amount of HA use (*x*-axis in Figure 3). Users had an average amount of HA use of 10.01 h, with a SD of 2.76 h (Coefficient of variation = 0.276). The middle 50% of users (*medium users*, between the first and third quartiles) ranged from 8.18 to 12.04 h (group mean = 10.16) of average HA use. The fact that the remaining 50% of the users exhibited an average HA use either below 8.18 (*light users*; group mean = 6.32) or above 12.04 (*heavy users*; group mean = 13.37) h indicates a substantial between-user variability.

Additionally, we investigated the within-user variability in the amount of HA use (*y*-axis in Figure 3). The average within-user SD was 3.88 h, indicating that the same user tended to use the hearing aids for varying durations throughout the logged days. A significantly larger within-user SD was observed for the medium users compared to both the light users (Two-sample *t*-test: *t* = 23.06, *p* < 0.001; Effect size: *d* = 0.44) and the heavy users (Two-sample t-test: *t* = 41.85, *p* < 0.001; Effect size: *d* = 0.81). This proves that both light users and heavy users were more consistent than medium users throughout the logged days (i.e., lower within-user SD) and constitutes an indication of users consistently displaying diverse behaviors in terms of HA use. The relationship between average HA use (*x*) and within-user SD (*y*) was modeled by fitting a second order linear regression model to the data. The line of best fit (*R*^2^ = 0.2) was described by the equation *y* = 0.91*x*−0.04*x*^2^. The maximum of the curve is around 10 h of HA use, indicating that the within-user SD increases with the amount of HA use for users using the HAs up to 10 h and it decreases for users using the HAs more than 10 h.

### Clustering Days of Hearing Aid Use

**Figure 4:**
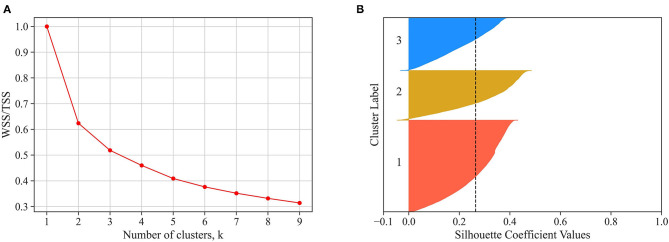
In **(A)**, the ratio of within sum of squares (WSS) to the total sum of squares (TSS) is displayed as a function of the number of clusters. The elbow plot suggests selecting three clusters since adding an additional cluster does not substantially increase the explained variation. In **(B)**, the Silhouette coefficient value of each observation is displayed for the three-cluster solution (i.e., for each cluster, the observations are ordered by their Silhouette coefficient value and displayed in ascending order as horizontal stacked lines). The average silhouette score is reported (dashed line). The three clusters have predominantly positive scores, suggesting valid clustering.

The substantial within-user variability in HA use suggests that a deeper analysis is warranted, which accounts for the hourly and daily variability in HA use. In addition to the amount of HA use, we also assessed patterns of HA use, defined as minutes of HA use per hour throughout the day. That was done by clustering the 453,612 logged days into typical days of HA use (see subsection Data Analysis). Based on the elbow method (Figure 4A), a three-cluster solution was chosen, which accounts for almost 50% of the variance among days. The Silhouette analysis (Figure 4B) indicated that the three clusters have predominantly positive scores, and there are no clusters with below-average silhouette scores.

**Figure 5:**
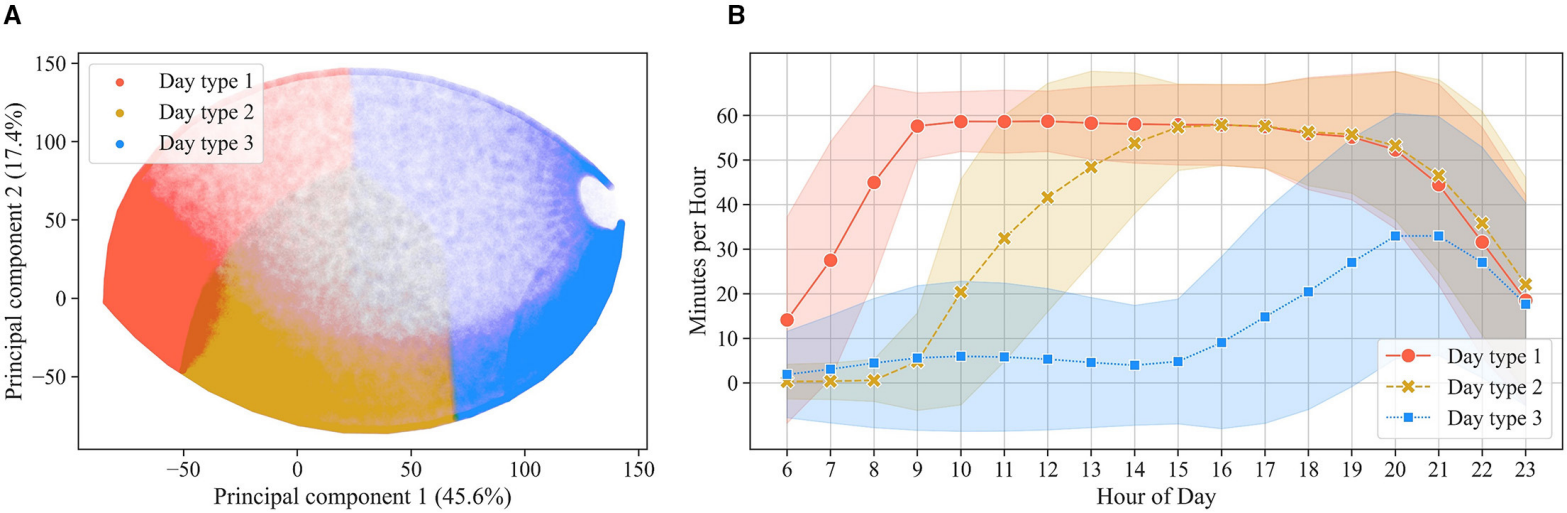
In **(A)**, the days of HA use are displayed as scatterplot against the two main principal components and colored by the three clusters (i.e., three day types). In **(B)**, the mean (±SD) of hourly HA use for each cluster is displayed.

Figure 5 displays the 453,612 days of HA use plotted by the two main principal components and colored by the three clusters. The eigenvectors suggest that the first principal component is negatively correlated with HA use in all hours of the day, differentiating between days of heavy use (Figure 5A, left) and days of light use (Figure 5A, right). The second principal component, instead, is positively correlated with HA use in the morning hours and negatively correlated with HA use in afternoon and evening hours, differentiating between days of morning HA use (Figure 5A, top) and days of HA use later in the day (Figure 5B, bottom). For each cluster, outliers were removed, resulting in 440,052 observations belonging to the three clusters. Looking at the hourly mean of HA use for each cluster (Figure 5B), it is possible to qualitatively evaluate the patterns underlying the clusters. Three distinct types of days of HA use can be identified: a full day of HA use (cluster 1, containing 204,062 days), a day of afternoon HA use (cluster 2, containing 120,810 days), and a day of sporadic evening HA use (cluster 3, containing 115,180 days).

A significant (*p* < 0.001), but negligible (Cramer's V = 0.05) association was found between the type of day of HA use and the day of the week (i.e., weekend vs. weekday). Full days of HA use occurred slightly (6%) more often during weekdays than during the weekend. Conversely, afternoon and sporadic evening days occurred slightly (4 and 1%) more often during the weekend than during weekdays.

### Clustering Users

**Figure 6:**
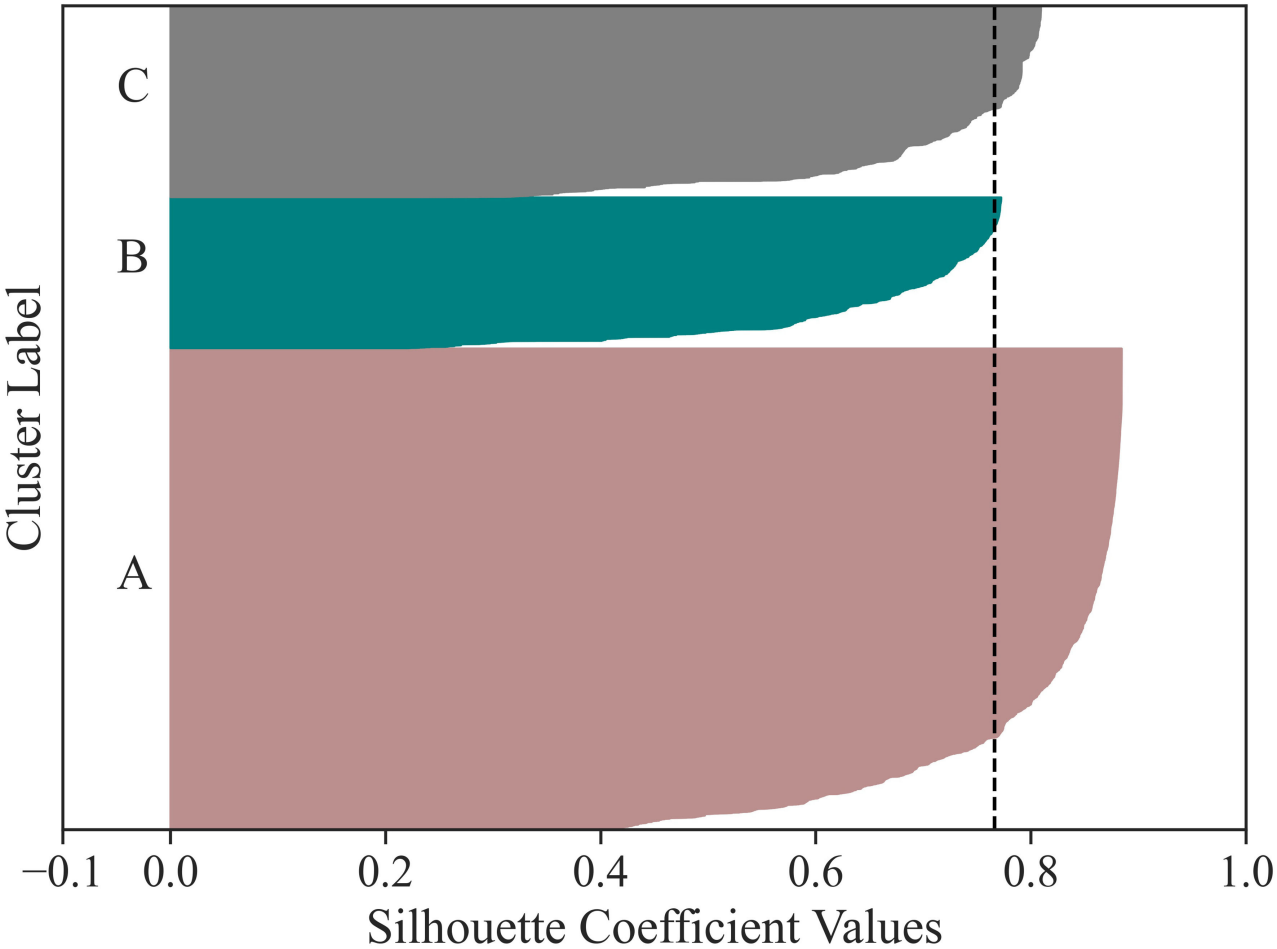
The Silhouette coefficient value of each observation is displayed for the three user clusters (i.e., for each cluster, the observations are ordered by their Silhouette coefficient value and displayed in ascending order as horizontal stacked lines). The average silhouette score is reported (dashed line). The three clusters have predominantly positive and large scores, suggesting valid clustering.

Having identified three types of days of HA use enables exploring HA user behavior, thus generating personalized insights, in a way that considers the day-to-day variation of each user. We explored the behavior of HA users by clustering the 15,905 users based on how they used the HAs during the logged days (see subsection Data Analysis). Each user is represented by the proportion of time spent in each of the three types of days of HA use. Four clustering techniques were evaluated. The optimal number of clusters for *k*-means and both HAC techniques were determined to be three. HDBSCAN also identified three clusters, with the minimum cluster size hyperparameter set to 1,000, in addition to considering some observations as noise. Based on three internal validation metrics, HDBSCAN was chosen (Table 1). The Silhouette analysis (Figure 6) suggested that the three clusters are of different sizes and have predominantly positive and large scores.

**Table 1:** Comparison of four different clustering techniques [K-means, HAC (Euclidean distance), HAC (Pearson correlation), and HDBSCAN] based on three internal validation metrics (Silhouette, Davies-Bouldin, and Caliñski-Harabasz).

	**K-means**	**HAC** **(Euclidean distance and Ward's method)**	**HAC** **(Pearson correlation and average linkage)**	**HDBSCAN** **(Pearson correlation)**
**Silhouette** (Higher is better)	0.4539	0.4264	0.6400	0.7604
**Davies-Bouldin** (Lower is better)	0.8267	0.7169	0.6001	0.4176
**Caliñski-Harabasz** (Higher is better)	18,473	13,732	35,802	70,327

*HDBSCAN is the best performing technique according to all three metrics*.

**Figure 7:**
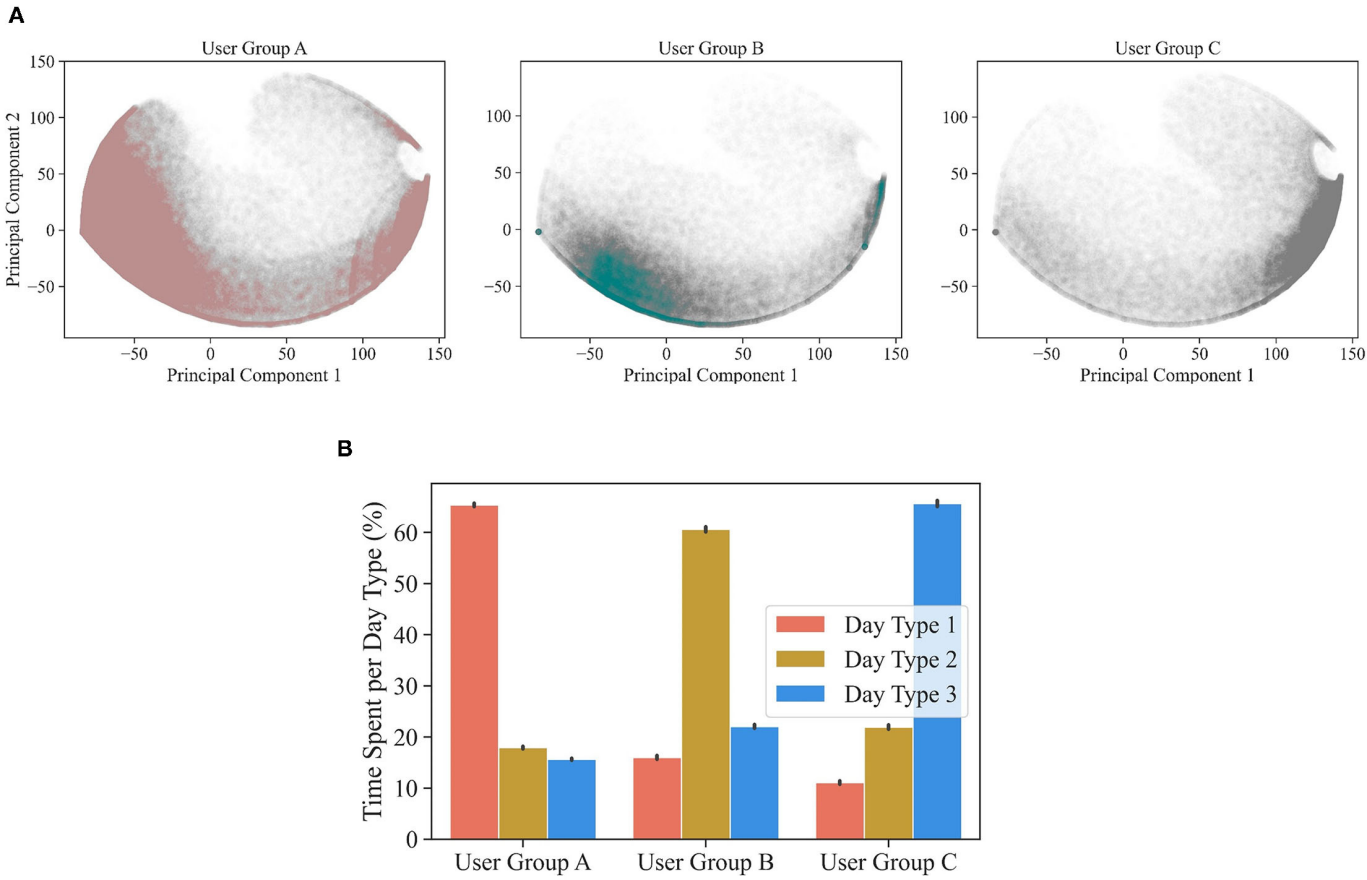
In **(A)**, for each of the three user clusters (i.e., user group A, B, and C), the days of HA use are displayed as scatter plot against the two main principal components. The distinct densities indicate that the three user groups experienced substantially different days of HA use. In **(B)**, the average proportion of time spent (±95% confidence interval) in each day type is displayed for each user cluster.

Figure 7A displays the days of HA use experienced by the users belonging to each user group. These plots can be directly compared with Figure 5A, which displays all days of HA use from all users. Each user group has a distinctive distribution of days. User group A is the largest cluster (7,862 users) and exhibits a higher density in the left corner of the figure, corresponding with day type 1 (i.e., full day of HA use). User group B (2,442 users) exhibits a higher density in the lower part of the figure, corresponding with day type 2 (i.e., day of afternoon HA use). User group C (3,148 users) has a higher density in the right corner of the figure, corresponding with day type 3 (i.e., day of sporadic evening HA use). Additionally, 2,453 users exhibited atypical behavior and were labeled as noise. The distinctive behavior of the three user groups is confirmed by their average time spent in each of the typical days of HA use (Figure 7B). User group A is predominantly having full days of HA usage, user group B is predominantly having days of afternoon HA use, user group C is predominantly having days of sporadic evening HA use. It should be noted that the predominant day of HA use is experienced around 60% of the time by the three user groups.

### Validating User Clustering Using Supervised Classifiers

We validated the HA user clustering by training an ensemble of three supervised classifiers (multiclass logistic regression, XGBoost and fully connected neural network) to predict the label of each user (user group A, B, C, or noisy user). The training input was the average day of HA use for each user, defined as minutes of HA use per hour throughout the day (from 6:00 to 23:59).

**Figure 8:**
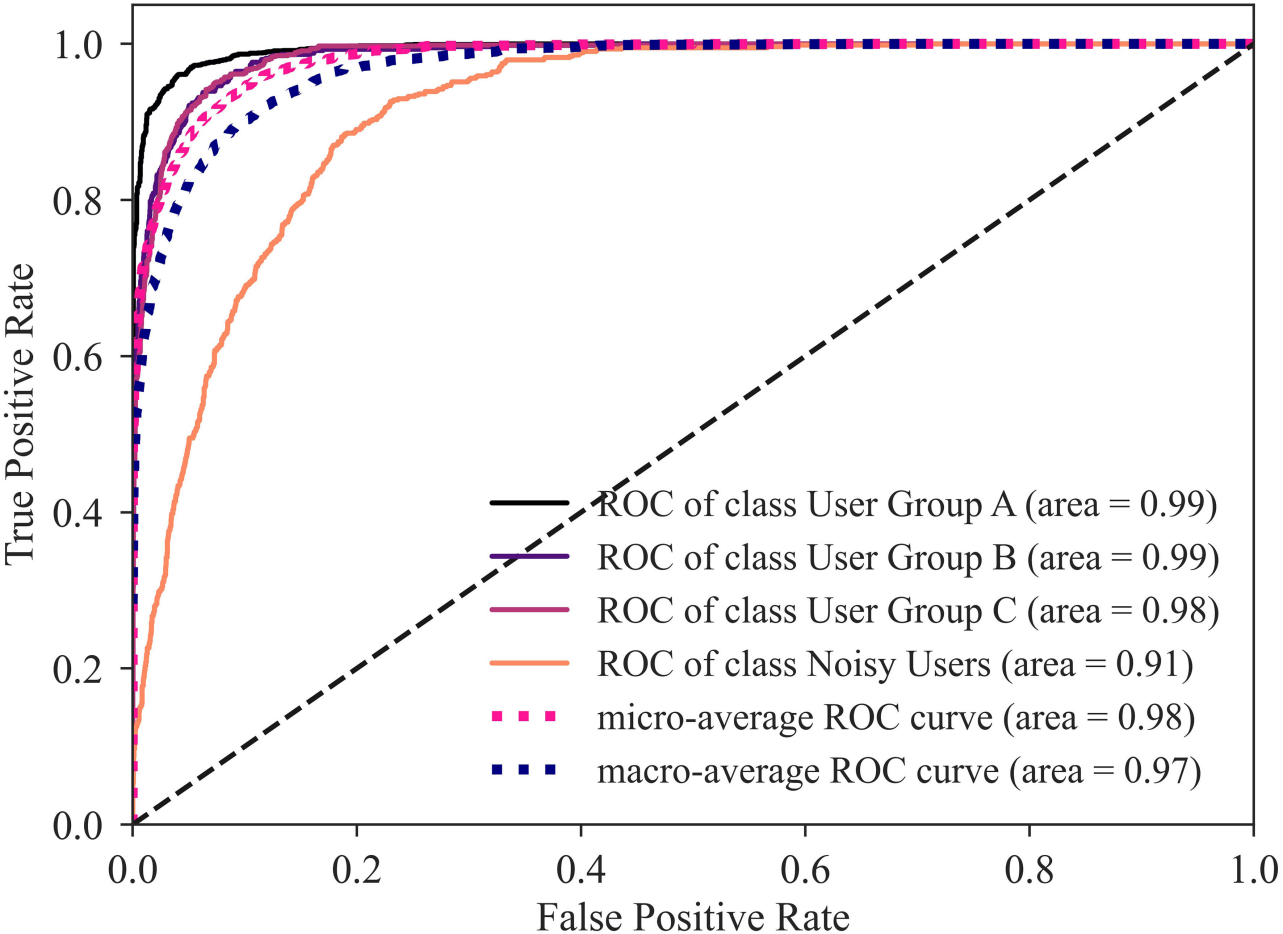
ROC-AUC plot for the ensemble of classifiers, illustrating the tradeoff between sensitivity (True Positive Rate) and specificity (False Positive Rate). The ideal point is the top-left corner, higher AUC is better. In this multiclass scenario, the individual classes are first binarized, the individual scores are computed for each user group, then micro-averages and macro-averages are calculated for each classifier.

When evaluating the three individual classifiers based on accuracy and ROC-AUC score (Table 2), XGBoost results to be the best performing classifier. In order to reduce bias, an ensemble of three supervised classifiers was defined. This simulates three artificial experts coming to a decision (40). The ensemble assigns each user to a group by majority voting between the three classifiers. In case where no majority could be defined, the group was decided by the best performing individual classifier (XGBoost). The ensemble accuracy was 86.04%, while the ROC-AUC score was 0.98. While the ensemble has a slightly worse accuracy than XGBoost, relying on classifiers from different classes mitigates the effect of bias that each classifier has. The ROC curves for the ensemble of classifiers (Figure 8) show that noisy users exhibiting atypical behavior are the most difficult to classify (i.e., lowest AUC). Conversely, the ensemble of classifiers successfully distinguishes between the three user groups.

**Table 2:** Comparison of three individual classifiers (multiclass logistic regression, XGBoost, and fully connected neural network) and of the classification ensemble based on two performance metrics (accuracy and ROC-AUC score).

**Classifier type**	**Accuracy** **(0-100 %)**	**ROC-AUC Score** **(micro-average)**
Logistic regression	81.51	0.97
XGBoost	87.08	0.98
FC neural network	85.56	0.98
Ensemble	86.04	0.98

*XGBoost is the best performing individual classifier according to both metrics*.

It is interesting to inspect the importance attributed by XGBoost to each of the 18 hours considered (Table 3). XGBoost values h9 and h15, indicating that these two hours are the ones that mostly differentiate the three user groups. This is consistent with the fact that each user group is characterized by a predominant day of HA use (Figure 7), and that h9 and h15 are the most effective hours in differentiating between the three day types (Figure 5B).

**Table 3:** Input feature importance returned by XGBoost.

**H6**	**H7**	**H8**	**H9**	**H10**	**H11**	**H12**	**H13**	**H14**	**H15**	**H16**	**H17**	**H18**	**H19**	**H20**	**H21**	**H22**	**H23**
0.013	0.014	0.028	**0.326**	0.017	0.019	0.021	0.039	0.064	**0.256**	0.042	0.037	0.021	0.025	0.024	0.014	0.017	0.015

*The values indicate how valuable each of the 18 features (from H6 in the morning to H23 at night) are in the construction of the boosted decision trees (internal to the model). The values greater than 0.2 are marked in bold. The model values divergence points between the three day types (H9, H15) (see Figure 5B). Total value is 1*.

## Discussion

While HA use has been traditionally assessed through subjective self-reports, smartphone-connected HAs enable objective data logging of HA use. This study investigates the objective HA use of a large cohort of HA users. 453,612 days of HA use logged by 15,905 users were analyzed.

The amount of HA use time is informative of how long the HA has been used during a day. On average, the users used the HAs for 10.01 h/day. This value is similar (11, 17) or slightly larger (10, 19) than previous studies objectively measuring HA use. When investigating the variability between users, this study found that 25% of users used the HAs for <8.18 h. This percentage is similar to a study by Laplante-Lévesque et al. (11), but smaller than other studies (12, 18, 19) objectively or subjectively assessing the amount of HA use of several users. The inclusion criteria of this study (i.e., users of the HearingFitness™ feature *via* a smartphone app) and the data cleaning criteria (i.e., days with at least 60 min of HA use) could explain the greater average HA use and the lower percentage of light users. Moreover, a greater average HA use could be explained by the fact that, for binaural HA users this study selected the larger value between the right and left ear. While HA users can either exhibit a low or high average amount of daily HA use, their day-to-day fluctuations in HA use provide a deeper understanding of HA use. The fluctuations in day-to-day HA use (i.e., within-user SD) were lower for light and heavy users compared to medium users, proving that a substantial number of users consistently displayed diverse behaviors in terms of HA use.

In addition to the amount of HA use, continuous data logging enables assessing how and when HAs were used during the day. Based on patterns of hourly use, the 453,612 days of HA use were clustered into three typical days. Forty-four percent of days were characterized by full HA use. This indicates that generally, when worn, HAs tend to be turned on in the morning (around 7), used uninterruptedly throughout the day, and turned off in the evening (around 22). Twenty-seven percent of days were characterized by afternoon use. This indicates that HAs are occasionally turned on in the late morning (around 11) and used uninterruptedly until the evening (around 22). This behavior might be due to a different individual daily rhythm or to a day encompassing different activities (e.g., weekend had a significant, but negligible effect on the day type). Twenty-six percent of days were characterized by sporadic evening HA use. This suggests that HAs are sometimes used in isolated occasions and for a limited number of hours. The remaining days (3%) were atypical days of HA use and exhibited infrequent behavior.

Based on the proportion of time spent in each of the typical days of HA use, the 15,905 users were clustered in three user groups. This method allowed to investigate users' behavior while preserving the individual day-to-day variability in HA use. Almost half of the users (group A, 49% of users) predominantly had full days of HA use. This group might include users that have an active life and engage in social interactions starting in the morning and throughout the entire day. Because of the inclusion criteria of this study (i.e., users of a smartphone app that tracks HA use), this group might be overrepresented. A smaller portion of users (group B, 15%) predominantly had days of afternoon use. This group might include users that engage in activities and social interactions later in the day. Group A and B, together, indicate that 64% of users tended to use the HAs uninterruptedly, a percentage similar to the 57% found by Laplante-Lévesque et al., (11). Twenty percent of users (Group C) predominantly had days of sporadic evening use. This group might either contain users that are not acclimatized to their HAs or users that do not depend on their HAs and only need them in specific situations (16). The remaining 15% of users were classified as noise, suggesting that some users have an uncommon behavior, more evenly alternating among the typical days of HA use. This percentage is in line with a study by Laplante-Lévesque et al., (11), according to which 23% of the subjects described their HA use to be different from day to day. Interestingly, in all three user groups, we found that the predominant day of HA use accounted for ~60% of the time, suggesting that users exhibited a substantial within-user variability in terms of day type experienced throughout the logged days. This aspect might not emerge from self-reported assessments that suffer from recall bias, as indicated by a previous study in which most participants (77%) reported their HA use to be the same every day (11).

The user clustering was validated by training a supervised classification ensemble to predict the cluster to which each user belongs. The high accuracy achieved by the supervised classifier ensemble (~86%) indicates valid user clustering. Indeed, this approach is based on the idea that good clustering should also support good classification, where the better the classification performance the higher the quality of the partition. As such, a high-quality partition is defined by compact clusters separated from each other to the extent that an artificial expert (i.e., a supervised classifier) can distinguish the cluster to which a new user belongs (39). This evaluation was performed to complement internal validation methods (i.e., using information of the clustering process). Internal validation methods attempt to evaluate cluster structure quality, the appropriate clustering algorithm, and the number of clusters without additional information but depend on assumptions such as the presence of underlying structure for each cluster, resulting in weaker results when they do not hold. Alternatively, cluster quality could theoretically be evaluated using external validation, which requires additional, “true” cluster labels to compare against. In real-world scenarios, finding “true” labels is often difficult as raw data may not have reference labels, thus making external validation methods unusable.

Clustering users based on their HA use patterns provides a deeper insight into the adoption of hearing care treatments and paves the way for more personalized solutions. For instance, users that predominantly have days of sporadic evening HA use might have specific needs compared to the users that uninterruptedly use the HA for the entire day. They might only need the HAs in specific situations and thus benefit from targeted HA settings or features. Additionally, training a supervised classifier based on data labeled by a clustering technique enables future predictions for new users. Based on the average day of HA use of a new user, the classifier can predict her user group, thereby identifying users with similar behaviors and potentially leveraging on the accumulated knowledge of existing users. This can improve the clinical flow by helping audiologists make data-driven decisions.

Looking into the future, a more advanced level of personalization could improve the quality of hearing care solutions and help alleviate major challenges concerning new users, such as the cold start problem. This can be defined as the delay between starting to use the HAs and the moment when enough data was generated locally for meaningful results. Furthermore, an individual's dynamic sound environment, or soundscape, may also be an important factor for personalization. Considering the large number of soundscapes a user may be exposed to throughout the day (public transport, social events, work environments, etc.), additional features can potentially account for both the within-user and the between-user variability. An effective clustering technique for grouping similar users may serve to balance this increase in complexity, especially if advanced privacy-preserving techniques such as federated learning and differential privacy are considered. Federated learning is a machine learning framework where models are trained locally, and afterwards aggregated between participating users. This type of model development could provide access to unrivaled amounts of quality user data, as privacy concerns can only be alleviated if users never have to give away their data. Real-world implementation of such a technique could provide tangible benefits to both existing users, as well as improve the experience of new users, thus enabling next-generation privacy focused personalization systems.

## Data Availability Statement

The data analyzed in this study is not publicly available. Requests to access the data should be directed to the corresponding author.

## Ethics Statement

In the sign-up process, the participants actively gave their consent for data to be collected, stored, and used for research purposes on aggregated levels. No personal identifier was collected. No ethical approval was required for this study according to Danish National Scientific Ethical Committee (26).

## Author Contributions

AP and T-IS conceived and designed the study, organized the database, and performed the statistical analysis. AP wrote the manuscript. JC, KJ, JL, NP, and KS supervised the findings and revised the final manuscript. All authors contributed to the article, read, and approved the submitted version.

## Conflict of Interest

AP, T-IS, and KJ are employed by Demant A/S. JC, NP, and KS are employed by Oticon A/S. The remaining author declares that the research was conducted in the absence of any commercial or financial relationships that could be construed as a potential conflict of interest.

## Publisher's Note

All claims expressed in this article are solely those of the authors and do not necessarily represent those of their affiliated organizations, or those of the publisher, the editors and the reviewers. Any product that may be evaluated in this article, or claim that may be made by its manufacturer, is not guaranteed or endorsed by the publisher.
